# Resting energy expenditure during spinal cord injury rehabilitation and utility of fat-free mass-based energy prediction equations: a pilot study

**DOI:** 10.1038/s41394-024-00682-x

**Published:** 2024-10-02

**Authors:** Amy N. Nevin, Sridhar S. Atresh, Angela Vivanti, Leigh C. Ward, Ingrid J. Hickman

**Affiliations:** 1 Department of Nutrition and Dietetics, Princess Alexandra Hospital, Brisbane, QLD Australia; 2 The Hopkins Centre, School of Health Sciences and Social Work, Griffith University, Brisbane, QLD Australia; 3 Faculty of Medicine, University of Queensland, Brisbane, QLD Australia; 4 Spinal Injuries Unit, Princess Alexandra Hospital, Brisbane, QLD Australia; 5 School of Human Movement and Nutrition Sciences, University of Queensland, Brisbane, QLD Australia; 6 School of Chemistry and Molecular Biosciences, University of Queensland, Brisbane, QLD Australia; 7 ULTRA Team, University of Queensland Clinical Trial Capability, Brisbane, QLD Australia

**Keywords:** Nutrition, Weight management

## Abstract

**Study design:**

Longitudinal observational study. Measurements were undertaken between weeks 4–6 post-spinal cord injury (SCI), repeated at week 8 and every 4 weeks thereafter until week 20 or rehabilitation discharge, whichever occurred first.

**Objectives:**

Observe variation in measured resting energy expenditure (REE) and body composition in males undergoing SCI rehabilitation, compare REE with SCI-specific prediction equations incorporating fat-free mass (FFM), and explore the prevalence of clinical factors that may influence individual REE.

**Setting:**

Spinal Injuries Unit, Brisbane, Queensland, Australia.

**Methods:**

Indirect calorimetry was used to measure REE and bioimpedance spectroscopy to assess body composition. Four SCI-specific FFM-based REE and basal metabolic rate (BMR) prediction equations were compared to measured REE. A clinically significant change in REE was defined as +/− 10% difference from the week 4–6 measurement. Clinical factors that may affect REE variations were collected including infection, pressure injuries, autonomic dysreflexia, spasticity, and medications.

**Results:**

Fifteen people participated (mean age 35 ± 13 years, 67% paraplegic). There was no statistically significant change in mean REE, weight, or body composition, and the Chun and Nightingale BMR prediction equations performed best (*r*_*c*_ > 0.8 at all time points). One-third of participants had >10% change in REE on 11 occasions, with clinical factors not consistently associated with the observed changes.

**Conclusion:**

During SCI rehabilitation, mean REE, weight, and body composition remain unchanged, and FFM-based BMR prediction equations may be an acceptable alternative to indirect calorimetry. Future research designs should avoid single indirect calorimetry measures as snapshot data may not represent typical REE in this population.

## Introduction

It is well established that total daily energy needs are reduced following spinal cord injury (SCI) [1]. This is largely because of reductions in resting energy expenditure (REE), which is the minimum amount of energy required to sustain the basic processes of life [2]. The reduction in REE following SCI is driven by alterations in body composition, specifically loss of fat-free mass (FFM) and concurrent increases in fat mass (FM) [3]. Although decreases in physical movement also contribute to lower daily energy requirements [4], REE is estimated to account for >80% of total daily energy needs in people with SCI [5]. For this reason, much of the research surrounding energy needs after SCI has focused on REE, which can be reliably measured using indirect calorimetry [6]. While the term REE is often used interchangeably with basal metabolic rate (BMR) in the SCI and wider literature, subtle differences in indirect calorimetry protocols do exist, with BMR requiring more stringent measurement conditions [7].

Energy requirements are thought to change over time following SCI. Catabolic conditions and physiological stress associated with acute injury are proposed to increase REE initially [8], followed by an obligatory loss in FFM and an associated decrease in REE [9, 10]. Without appropriate dietary modification, weight gain then ensues, leading to high rates of neurogenic obesity and cardiometabolic disease [11–13]. Due to limited studies of REE and body composition in the first six months following SCI [1], the timing of these changes remain poorly understood. The only study to longitudinally monitor REE during this period was conducted by Felleiter et al. in 2017 [14]. Mean REE was reduced by 19% between week 2 and week 130 post-injury, including a reduction of 10.5% between week 2 and 6 [14]. As results are yet to be replicated in other studies, the implications for clinical practice and research remain unclear.

Lack of knowledge surrounding energy needs and how they change have been identified by people with SCI as barriers to healthy eating [15]. Accurate assessment and prescription of energy requirements early in the SCI trajectory is therefore key and should form the foundation of specialised nutrition care. Currently, clinicians are unable to accurately prescribe energy requirements without the use of an indirect calorimeter, as traditional prediction equations overestimate energy needs in people with SCI [1]. Since indirect calorimetry is expensive and unavailable in many settings, research efforts have focused on developing and validating SCI-specific REE and BMR prediction equations, many of which incorporate measures of FFM [9, 16–18]. Although preliminary findings indicate greater accuracy compared to traditional prediction methods [17, 19], these new equations are derived from single measures of REE or BMR and FFM in people with chronic SCI and are yet to be validated in a longitudinal cohort undergoing rehabilitation.

There is limited understanding of how clinical factors influence REE in people with SCI. This is especially relevant in acute and rehabilitation environments where the clinical condition may be more dynamic, making the interpretation of single indirect calorimetry measures more difficult. Evidence from non-SCI populations supports the impact of multiple medications on REE [20–23]. Spasticity is also proposed to alter REE, although studies have failed to confirm a direct relationship [24, 25]. Fever and infection were associated with increased REE in people with chronic SCI, whereas pressure injuries and subsequent surgical repair were not [26]. As many of these clinical factors have precluded participation in studies of REE [14, 24, 27], a greater understanding of their impact is needed to determine the broader applicability of SCI-specific REE or BMR prediction equations and explore whether the development of injury factors is required.

The primary aim of this pilot study was to observe REE and body composition in males undergoing SCI rehabilitation and compare measured REE with SCI-specific REE prediction equations that incorporate measures of FFM. The secondary aim was to investigate the prevalence of clinical factors that may influence REE at the individual level during rehabilitation and explore implications for clinical practice and future research.

## Methods

### Participants

Males with a recent SCI admitted to the Spinal Injuries Unit at the Princess Alexandra Hospital for rehabilitation were eligible to participate in the study. Exclusion criteria included age <18 years, inability to provide informed consent, >6 weeks post-injury, an expected rehabilitation admission of <8 weeks, American Spinal Injury Association Impairment Scale (AIS) D injuries, cardiac pacemaker, tracheostomy, ventilator dependence, reliance on supplemental oxygen, halo brace or a decision from the treating rehabilitation specialist that the person was unsuitable to participate.

### Study design

This was a longitudinal observational pilot study. Data collection commenced at a time between weeks 4 and 6 post-injury (week 4–6 measurement), depending on the timing of admission to the rehabilitation unit. Data collection was repeated at week 8 and continued every four weeks until rehabilitation discharge or week 20, whichever occurred first.

### Demographic and clinical data

Electronic medical records were used to collect age (years) on admission, comorbidities, injury mechanism (traumatic vs non-traumatic), and SCI level according to the International Standards for Neurological Classification of Spinal Cord Injury [28] at rehabilitation admission and discharge. To assist with the interpretation of REE values, the following clinical information was recorded on each occasion of data collection: presence of a pressure injury > stage 2 according to international staging guidelines [29] or other wounds (yes/no), surgery within the last seven days (yes/no), infection within the last seven days (yes/no), autonomic dysreflexia within 24 h pre- or post-data collection (yes/no), temperature >38 degrees celsius within 7 days pre- or 48 h post-data collection (yes/no), spasticity (yes/no), change in medications that may impact REE since the last occasion of data collection (new or ceased, increased or decreased dose) including beta blockers, alpha-agonists, opioids, thyroid agents, sympathomimetic amines (e.g. pseudoephidrine), glucocorticoids or muscle relaxants (yes/no) [20–23].

### Anthropometry

Height was assessed at the week 4–6 measurement by measuring supine bed length to the nearest 0.5 cm (Lufkin Executive diameter W606PM, Maryland, USA) [30]. Body weight was measured in the morning, within 24 h of all data collection using a ceiling hoist scale (Arjo Scale 2016, Macquarie Park, Australia) or wheelchair scale (Wedderburn WM501, Ingleburn, Australia) to the nearest 0.1 kg, in the morning. To calculate body mass index (BMI) at weeks 4–6, weight (kg) was divided by height (m) squared.

### Resting energy expenditure

Indirect calorimetry was used to measure REE (True One 2400 Metabolic Measurement System, ParvoMedics, UT). The protocol adhered to best practices [6] and has been used successfully in people with SCI in a rehabilitation setting [26, 31]. Individuals were tested in a thermoneutral environment, at their bedside in the SCI rehabilitation unit, on waking following an overnight fast (≥8 h without food, caffeine, alcohol or nicotine), prior to administration of any medications, ≥14 h since any physical activity, resting quietly for 20 min prior to testing. Gas and volume calibration occurred before each measurement as per manufacturer instructions. A ventilated canopy hood measured the volume of inspired oxygen (VO_2_) and expired carbon dioxide (VCO_2_) for at least 15 min while participants remained still, lying supine in bed. The calorimeter software calculated REE at regular intervals according to the Weir equation [32]. Excluding the first 5 min of data, an average REE value was calculated for each measurement using a 5–10 min period with a coefficient of variation for VO_2_ and VCO_2_ of <10% to reflect steady-state REE [6]. If steady state REE was not achieved (n = 5/57 measurements), an average REE value was calculated using a 5 min period with the lowest coefficient of variation for VO_2_ and VCO_2_. If this REE value was within 10% of the participants’ previous and/or following measurement, the REE data were retained and included for analysis. Although this protocol adheres to conditions required for measurement of BMR given individuals was tested at their bedside, immediately on waking (instead of after travelling to a testing centre, as per the definition of REE by Alazzam and Gorgey 2023 [7]), data collection occurred in an active hospital ward setting, behind closed curtains in shared rooms, making factors such as lighting and noise difficult to control. As the impact of noise and lighting alone on metabolic rate is unclear, data is described as REE rather than BMR. Given REE is known to vary by up to 10% in healthy individuals, a clinically significant change was defined as a difference of >10% compared to the week 4–6 measurement [6].

### Body composition

Body composition was measured immediately following indirect calorimetry using a four-terminal bioimpedance spectroscopy (BIS) instrument (SFB7, ImpediMed Ltd., Brisbane, Australia). The protocol used was consistent with other SCI studies and has been described in depth elsewhere [33, 34]. Briefly, skin surface Ag/AgCl ECG-style measurement electrodes (3M Red Dot 2330, 3M, Sydney, Australia) were positioned between the medial and lateral malleoli at the ankles, the head of the radius and ulna at the wrists, and at the base of the fingers and toes while participants were lying supine in bed and after each site was cleaned with alcohol. Whole body impedance measurements were recorded on both the right and left sides. Measurements were obtained in quintuplicate and analysed using Bioimp version 4.18.0 software (Impedimed Ltd., Brisbane, Australia), with mean values of resistance at 50 kHz used in subsequent calculations of body composition. Whole body composition (FFM and FM in kg) was calculated using the Kocina and Heyward equation [35] for the right and left body. Mean FFM and FM values were then calculated for each time point and included for analysis.

### Comparison with energy prediction equations

Four SCI-specific REE or BMR prediction equations incorporating FFM values were identified [9, 16–18] (Table 1). Each equation was used to predict participants’ REE at each time point for comparison with measured REE. A conversion factor of 0.239 was used to convert kj to kcal.

**Table 1:** Resting energy expenditure and basal metabolic rate prediction equations incorporating measures of body composition used for comparison against measured resting energy expenditure.

Author	Population	Equation
Buchholz et al. [9]	n = 28 • 61% male • 100% paraplegia • 64% motor complete • Time since injury 11 ± 10 years	REE (kj/day) = 10682 - 1238(ln age) - 521(sex) - 24(H) + 87(FFM)
Chun et al. [16]	n = 50 • 76% male • 46% paraplegia • 100% motor complete • Time since injury: 12 ± 7 years	BMR (kcal/d) = 24.5 x FFM + 244.4
Nightingale and Gorgey [17]	n = 30 • 100% male • 70% paraplegia • 94% motor complete • Time since injury: 9 ± 9 years	BMR (kcal/d) = 23.469 x FFM + 294.330
Ma et al. [18]	n = 48 • 63% male • 65% paraplegia • 52% motor complete • Time since injury: 17 ± 13 years	REE (kcal/d) = 925.571 + 14.648 x FFM

*REE* resting energy expenditure, *BMR* basal metabolic rate, *Age* age in years, *ln* log-transformed, *Sex* 0 for males and 1 for females, *H* height in centimeters, *FFM* fat-free mass in kilograms.

### Statistical analysis

Similar to previous nutrition-related pilot studies of people with SCI [26], the target number of participants was n = 20. Normality was assessed using the Shapiro-Wilk test and visual inspection of box plots. To investigate whether REE, weight or body composition (FFM, FM) changed over time relative to other data collection points, a linear mixed model with a fixed effect for time (modelled as a categorical variable; weeks 4–6 was the reference level) and a random intercept for each subject was used, with adjustments for multiple testing made using Dunnett’s method. Lin’s concordance correlation coefficient was used to compare measured REE with predicted REE, where concordance was considered good if *r*_*c*_ > 0.6 and high if *r*_*c*_ > 0.8 [36]. The median absolute percentage error, mean bias and limits of agreement (mean ± two standard deviations) were calculated as described previously [37]. Statistical analyses were performed using SPSS Version 27 (IBM Corp., Armonk, NY). All normally distributed data are presented as mean ± standard deviation, and median (interquartile range) for non-normally distributed data. Categorical variables are presented as count (percentage). Significance was set at *p* < 0.05.

## Results

Fifteen eligible participants took part in the pilot study. The target of n = 20 was unable to be reached due to significant recruitment challenges, including those related to the COVID-19 pandemic (see supplementary information for recruitment flowchart). Demographic data is presented in Table 2. Neurological level of injury spanned from C2 to L1, and participants’ ages ranged from 20–61 years (median age = 31 years). Three participants with AIS C injuries were reclassified as AIS D after collection of week 4–6 data had occurred but were retained in the study. Four individuals regained the ability to walk toward the end of their rehabilitation (n = 1 independently, n = 3 with aids), with the remaining 11 participants requiring a wheelchair for all mobility on discharge. Two participants had a history of hypertension, with no other chronic diseases reported.

**Table 2:** Participant demographic data (n = 15).

	Mean ± SD or n (%)
**Age at injury** (years)	35 ± 13
**Body mass index** (kg/m^2^)	27.1 ± 7.0
**Injury mechanism**	
Traumatic	11 (73)
Non-traumatic	4 (27)
**Injury level**	
Tetraplegia	5 (33)
Paraplegia	10 (67)
**Admission AIS**	
A or B (motor complete)	6 (40)
C	6 (40)
D	3 (20)
**Discharge AIS**	
A or B (motor complete)	5 (33)
C	4 (27)
D	6 (40)
**Number of weeks since injury at time of rehabilitation discharge**	21.7 ± 7.3

*AIS* American Spinal Injury Association Impairment Scale.

**Fig. 1:**
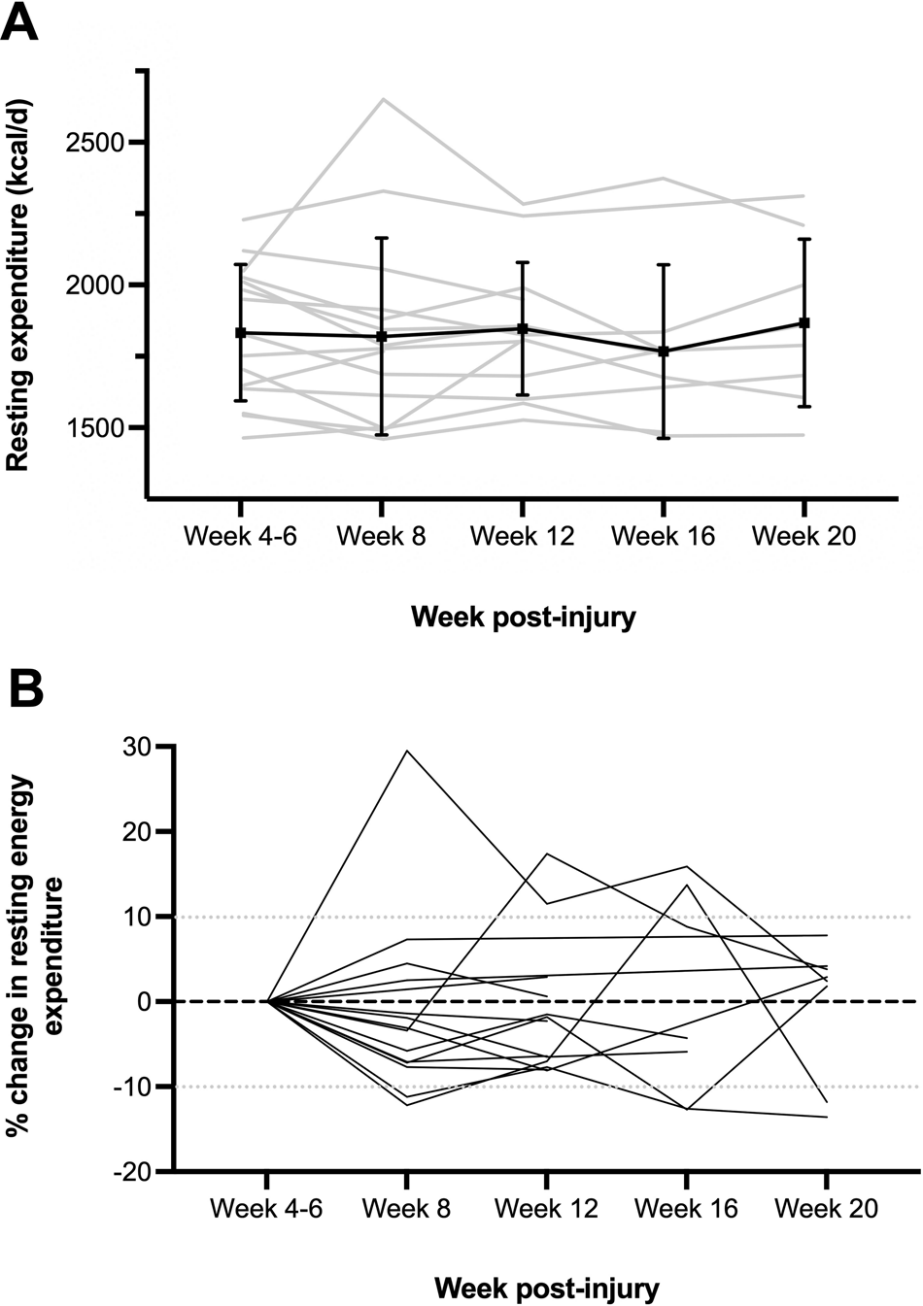
Change in resting energy expenditure (REE) throughout rehabilitation. **A** Mean REE at each time point. Error bars represent the standard deviation and grey lines represent the REE of individual participants. **B** Percentage change in each participant’s REE at each time point compared to the week 4–6 measurement. The dotted grey line represents +/– 10% of the week 4–6 REE measurement, where a change >10% is considered clinically significant.

Data collection occurred on 57 occasions, with n = 4 participants missing one episode each due to being medically unwell or the indirect calorimeter undergoing repairs. Figure 1 displays the mean REE at each time point (1A) and the percentage change in each participants’ REE compared to the week 4–6 measurement (1B). At the group level, there was no clinically significant (>10% increase or decrease) or statistically significant change in mean REE for any time point comparison (*p* > 0.05). At the individual level, two-thirds of participants remained within 10% of their week 4–6 REE measurement throughout the duration of their rehabilitation. The remaining five participants were observed to have a clinically significant change in their REE on a total of eleven occasions. The change in REE for these participants ranged from a decrease of 13.6% to an increase of 29.5% (Fig. 1B).

**Fig. 2:**
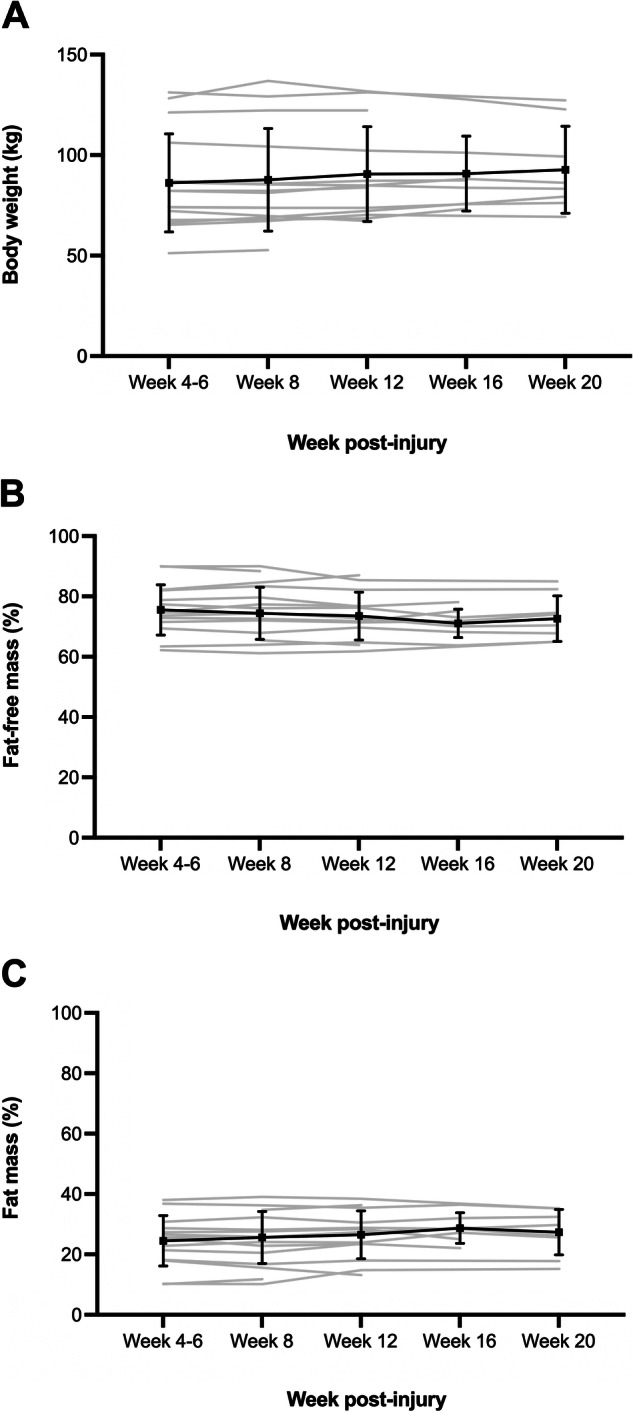
Change in body weight and body composition during rehabilitation. **A** Body weight, **B** fat-free mass % and **C** fat mass %. Error bars represent the standard deviation and grey lines represent the data of individual participants.

Figure 2 demonstrates mean weight and % body composition (FM, FFM). No statistically significant change was identified at any time point when compared to the week 4–6 measurement (*p* > 0.05 for all time point comparisons). Table 3 summarises the concordance agreements, mean bias, limits of agreement and median absolute percentage error between measured REE and four FFM-based REE or BMR prediction equations. The Chun et al. and Nightingale and Gorgey equations consistently showed the highest concordance agreements (*r*_*c*_ > 0.8), lowest limits of agreement (<20%) and smallest median absolute percentage error (2.5 – 8.6%).

**Table 3:** Agreement summary for resting energy expenditure and basal metabolic rate prediction equations compared to measured resting energy expenditure throughout spinal cord injury rehabilitation.

Week post injury		4–6 (n = 14)	8 (n = 14)	12 (n = 13)	16 (n = 7)	20 (n = 8)
**Resting energy expenditure (mean, SD)**		1833 ± 239	1820 ± 345	1847 ± 232	1767 ± 304	1867 ± 293
**Buchholz et al**. [9]	Lins *r*_*c*_^a^	0.554	0.786	0.747	0.759	0.825
	95% CI	0.069, 0.827	0.569, 0.901	0.364, 0.914	0.170, 0.948	0.442, 0.954
	Mean bias and LOA (kcal/d)^b^	40 (−372, 452)	13 (−357, 383)	26 (−287, 339)	−37 (−408, 334)	−3 (−303, 298)
	Mean bias and LOA (%)^b^	2 (−20, 25)	1 (−20, 21)	14 (−16, 18)	2 (−23, 19)	0 (−16, 16)
	MAPE (%)^c^	8.1	9.4	4.4	10.6	7.6
**Chun et al**. [16]	Lins *r*_*c*_^a^	0.838	0.881	0.846	0.823	0.874
	95% CI	0.589, 0.941	0.702, 0.955	0.603, 0.946	0.408, 0.956	0.547, 0.970
	Mean bias and LOA (kcal/d)^b^	60 (−188, 308)	21 (−276, 319)	9 (−220, 239)	−43 (−344, 258)	1 (−263, 265)
	Mean bias and LOA (%)^b^	3 (−10, 17)	1 (−15, 18)	0.48 (−12, 13)	−2 (−19, 15)	0 (−14, 14)
	MAPE (%)^c^	4.2	6.7	2.5	8.6	3.1
**Nightingale and Gorgey** [17]	Lins *r*_*c*_^a^	0.825	0.868	0.879	0.820	0.863
	95% CI	0.568, 0.936	0.685, 0.948	0.657, 0.961	0.418, 0.953	0.567, 0.935
	Mean bias and LOA (kcal/d)^b^	73 (−167, 313)	37 (−267, 340)	26 (−196, 248)	−27 (−333, 280)	19 (−249, 287)
	Mean bias and LOA (%)^b^	4 (−9, 17)	2 (−15, 19)	1 (−11, 13)	−2 (−19, 16)	1 (−13, 15)
	MAPE (%)^c^	4.7	6.2	3.3	7.8	3.9
**Ma et al**. [18]	Lins *r*_*c*_^a^	0.793	0.702	0.793	0.598	0.700
	95% CI	0.582, 0.904	0.533, 0.817	0.572, 0.907	0.256, 0.808	0.426, 0.856
	Mean bias and LOA (kcal/d)^b^	−10 (−257, 236)	−35 (−441, 371)	−31 (−267, 206)	−83 (−473, 306)	−28 (−376, 320)
	Mean bias and LOA (%)^b^	−1 (−14, 13)	−2 (−24, 20)	−2 (−14, 11)	−5 (−27, 17)	−1 (−20, 17)
	MAPE (%)^c^	6.2	5.9	5.7	8.3	6.3

^a^Lin’s concordance correlation coefficient.

^b^Mean bias (measured REE – prediction equation REE) and limits of agreement (LOA, mean ± two standard deviations).

^c^Median absolute percentage error.

The prevalence of clinical factors that may impact REE are presented in Table 4. Spasticity, medication changes and infection were the most common. While these clinical factors were associated with some of the significant changes to REE observed among individuals, the impact was inconsistent. For example, a sustained decrease in REE (11–13%) was observed in one participant requiring increasing numbers and dosage of muscle relaxants due to spasticity, whereas REE remained unchanged for others requiring an increase in the same medications. Another participant had a 29.5% increase in REE coinciding with a skin infection, whereas other participants’ REE remained stable despite urinary tract infections (further examples demonstrated in supplementary information).

**Table 4:** Prevalence of clinical factors that may impact resting energy expenditure at each time point, number of participants, n (%).

Week post injury	4–6 (n = 15)	8 (n = 14)	12 (n = 13)	16 (n = 7)	20 (n = 8)
**Febrile episode** (7 days prior or 48 h post)	1 (7)	1 (7)	0 (0)	0 (0)	0 (0)
**Autonomic dysreflexia** (24 h pre and post)	0 (0)	0 (0)	0 (0)	0 (0)	0 (0)
**Surgical procedure** (7 days prior)	0 (0)	0 (0)	0 (0)	0 (0)	1 (13)
**Infection** (7 days prior)	5 (33)	4 (29)	1 (8)	0 (0)	2 (25)
**Pressure injury** **≥** **stage 2 or other wounds**	5 (36)	0 (0)	0 (0)	0 (0)	0 (0)
**Spasticity**	7 (26)	9 (65)	9 (62)	7 (100)	8 (100)
**Smoking**	0 (0)	1 (0)	2 (15)	1 (14)	1 (13)
**Change in medications that may impact resting energy expenditure since last time point**	-	7 (50)	9 (69)	2 (29)	6 (75)

## Discussion

This is only the second study to longitudinally monitor REE and body composition in people with a recent SCI, and the first to explore the accuracy of FFM-based REE and BMR prediction equations in the SCI rehabilitation setting. There were no significant changes to mean REE, weight or body composition between weeks 4 and 20 of injury. The BMR prediction equations developed by Chun et al. [16] and Nightingale & Gorgey [17] performed well against measured REE during the rehabilitation admission. Clinically significant spikes or drops in REE were observed at various time points in one-third of individuals yet could not consistently be explained by accompanying clinical data.

The finding that mean REE, body weight, and body composition remained stable during SCI rehabilitation is consistent with the Felleiter study, where no changes in REE, weight or body composition were observed between week 6 and week 26 post-injury [14]. In that study, statistically significant reductions in these parameters were only observed when compared to measures undertaken earlier (week 2) and later (week 130) than in the present study. Mean REE was higher in this study, most likely due to differences in participant demographics. Unlike the Felleiter study, participants with acute complications (such as active infections) or a BMI > 30 kg/m^2^ were included. This, combined with a greater proportion of participants with incomplete (AIS C or D) injuries and a male-only cohort, likely explains the higher REE values. Other studies have failed to detect marked changes in mean weight or body composition assessed by bioimpedance during SCI rehabilitation [38, 39]. This reiterates that the initial obligatory negative nitrogen balance and associated loss of FFM occurs rapidly after SCI [10] and may begin to stabilise in early rehabilitation.

Consistent with a recent study conducted in 26 people with chronic SCI (n = 20 males, 65% paraplegia, 67% AIS A) [19], the BMR prediction equations from Chun et al. 2017 [16] and Nightingale and Gorgey 2018 [17] performed best in this study. This may be due to similarities in participant demographics, with both studies including predominantly males of a similar age and FM % to the present study. Consistencies in indirect calorimetry protocols may also be relevant. In contrast, the use of a mouthpiece rather than a canopy hood in the Ma et al. study may have impacted the REE data and subsequent prediction equation performance given the recognised discomfort associated with the mouthpiece device [40]. The presence of clinical factors may have also impacted measured REE or BMR in all studies, yet these are often poorly described or may not have been detected at all. While REE and BMR prediction equations continue to show promise, there is a need to consider what level of precision is pragmatic for clinical application. In addition to a low mean bias and median absolute percentage error, limits of agreement ±10% of measured REE or BMR may be an appropriate target considering the known variability in REE and BMR [6]. Determining SCI-specific activity factors also requires further exploration to support the transformation of predicted REE and BMR into estimates of total daily energy requirements.

This study paid meticulous attention to capturing clinical data that may impact REE in people with SCI. Although these were not consistently associated with the significant changes in REE observed in some individuals, routine monitoring and reporting is recommended as part of future study protocols to support accurate interpretation of indirect calorimetry data. Findings from this work question the representativeness of single indirect calorimetry measures, which historically have been used to assess the impact of interventions in clinical trials [41, 42] and to develop FFM-based REE or BMR prediction equations [9, 16–19]. Future research should consider a minimum of two measures undertaken within 1-2 weeks, with a third undertaken if the difference exceeds 10%. Similarly, for REE to be selected as an outcome measure in clinical trials, the intervention tested should be expected to achieve >10% difference in baseline REE or BMR to be confident of a change outside of daily variation. This study also indicates that the frequency of longitudinal indirect calorimetry assessments after SCI could be rationalised. However, the inclusion of event-triggered additional assessments (for example, in the case of fever, infection, and/or changes to relevant medications such as muscle relaxants) embedded in study protocols would assist with building the evidence base surrounding clinical factors that may impact REE in people with SCI.

### Limitations

This pilot study has several limitations. Like other longitudinal SCI research, this study faced significant recruitment challenges. Although those related to the COVID-19 pandemic were difficult to overcome, a changing SCI demographic and delays in rehabilitation admission required multiple evidence-based strategies to address (supplementary information) [43]. Many SCI studies are burdened by incomplete recruitment which compromises success, and the use of tools such as the SEAR framework and QRI-2 are recommended if recruitment targets are not being met [44, 45]. The decision to include people with non-traumatic SCI, incomplete injuries (AIS D) and a higher BMI may have increased heterogeneity in the outcome measures. When combined with the small sample, this heterogeneity also prevented meaningful analysis of SCI subgroups (such as complete vs incomplete SCI, or paraplegia vs tetraplegia) for all outcome measures, including prediction equation performance. However, it also increases generalisability of study findings given these characteristics are reflected in SCI populations worldwide [46–48]. The inclusion of males only is also a limitation. Future longitudinal studies should include female participants but carefully consider the timing of indirect calorimetry measurements given the potential for menstruation to confound REE [49]. Finally, the use of BIS to assess body composition may be considered a limitation. There is conflicting evidence regarding the accuracy of BIS compared to other methods such as the 4-compartment model, deuterium dilution and dual x-ray absorptiometry in people with SCI [33, 34, 50]. However, BIS was the most practical and accessible method available given the longitudinal nature of the study, and findings suggest that FFM-based BMR prediction equations still perform well when BIS is used to assess body composition.

## Conclusion

Mean REE, weight, and body composition remained stable throughout SCI rehabilitation, and FFM-based BMR prediction equations could be an acceptable alternative to indirect calorimetry in this setting. Clinical factors that may impact REE were prevalent among participants but were not consistently associated with clinically significant changes observed in some individuals’ REE. Single indirect calorimetry measurements may not accurately represent typical REE in this population and this should be considered when designing study protocols in future.

## Supplementary information

Supplementary information

## Data Availability

Deidentified data described in the manuscript is available in supplementary information. Requests for additional deidentified data will be made available upon request pending local ethical approval.
