# Supplementary material for: Resting energy expenditure during spinal cord injury rehabilitation and utility of fat-free mass-based energy prediction equations: a pilot study
Source: Spinal Cord Ser Cases. 2024 Oct 2;10:70. doi: 10.1038/s41394-024-00682-x (PMC11447238; doi:10.1038/s41394-024-00682-x)

Supplementary information

Table 1 - Resting energy expenditure (REE), weight, body composition (fat-free mass, fat mass) and medication changes that may impact REE throughout spinal cord injury rehabilitation. Clinically significant changes to REE (>10% compared to the week 4-6 measurement) are highlighted in yellow. AIS = American Spinal Injury Association Impairment scale.

Participant ID		1	2	3	4	5	6	7	8	9	10	11	12	13	14	15
Age		44	51	24	21	21	61	26	31	20	36	30	25	40	48	54
Admission AIS		T4 C	C4 D	C2 C	T9 D	C7 B	T2 A	T4 A	T2 C	T10 C	T5 C	T4 A	C4 B	T3 C	C3 C	L1 A
Discharge AIS		T3 D	C2 D	C3 D	T9 D	C7 C	T2 A	T6 A	T4 D	T10 C	T5 C	T4 A	C4 B	T3 D	C6 B	L1 C
	Week															
**REE (kcal/d)**	4-6	2231	1541	1462	1752	1635	2122	1951	2051	1647	1548	1827	2027	1703	2012	1983
	8	2332	1488	1498		1612	2057	1914	2656[2]	1767	1458	1686	1881	1495[4]	1787	1843
	12	2244	1809[1]		1803	1598	1950	1825	2286		1525	1680	1991	1584	1857	1856
	16		1676					1835	2377[2]		1482		1770[3]	1469[4]	1758	
	20	2315	1605			1682		2000	2212			1860	1788[3]	1472[4]		
**Weight (kg)**	4-6	131.0	73.8	51.0	72.0	65.0	121.0	82.0	128.0	73.9	67.5	66.2	106.0	86.2	86.8	82.0
	8	128.9	73.5	52.5		67.0	122.0	81.0	136.7	73.6	67.7	68.8	104.0	85.2	85.5	82.0
	12	131.0	73.5		67.0	70.0	122.0	84.6	131.5		68.3	72.0	102.0	84.6	87.0	83.6
	16		75.4					87.9	127.5		73.0		101.0	83.5	87.2	
	20	127.0	76.0			69.0		86.0	122.5			79.1	99.0	83.0		
**Fat-free mass (kg)**	4-6	80.9	54.8	45.8	58.9	58.2		63.1	80.7	54.1	52.9	54.0	73.1	61.3	62.9	61.1
	8	78.4	54.5	46.2		60.0	79.4	61.3	86.9	54.5	53.7	57.1	70.2	61.1	61.7	63.1
	12	80.4	53.2		58.0	59.5	77.5	64.2	84.7		52.1	58.9	70.7	60.1	62.1	63.9
	16		52.5					63.8	80.8		56.7		68.5	59.6	65.2	
	20	82.1	53.2			58.4		63.8	79.1			64.9	66.7	61.2		
**Fat mass (kg)**	4-6	50.1	19.0	5.2	13.1	6.8		18.9	47.3	19.8	14.6	12.2	32.9	24.9	23.9	20.9
	8	50.5	19.0	6.3		7.0	42.6	19.7	49.8	19.1	14.0	11.7	33.8	24.1	23.8	18.9
	12	50.6	20.3		9.0	10.5	44.5	20.4	46.8		16.2	13.1	31.3	24.5	24.9	19.7
	16		22.9					24.1	46.7		16.3		32.5	23.9	22.0	
	20	44.9	22.8			10.6		22.2	43.4			14.2	32.3	21.8		

**Fat-free mass (%)**	4-6	61.8	74.3	89.6	81.8	89.5		77.0	63.0	73.2	78.4	81.6	69.0	71.1	72.5	74.5
	8	60.8	74.1	88.0		89.6	65.1	75.7	63.6	74.0	79.3	83.0	67.5	71.7	72.2	77.0
	12	61.4	72.4		86.6	85.0	63.5	75.9	64.4		76.3	81.8	69.3	71.0	71.4	76.4
	16		69.6					72.6	63.4		77.7		67.8	71.4	74.8	
	20	64.6	70.0			84.6		74.2	64.6			82.0	67.4	73.7		
**Fat mass (%)**	4-6	38.2	25.8	10.4	18.2	10.5		23.0	37.0	26.8	21.6	18.4	31.0	28.9	27.5	25.5
	8	39.2	25.9	12.0		10.4	34.9	24.3	36.4	26.0	20.7	17.0	32.5	28.3	27.8	23.0
	12	38.6	27.6		13.4	15.0	36.5	24.1	35.6		23.7	18.2	30.7	29.0	28.6	23.6
	16		30.4					27.4	36.6		22.3		32.2	28.6	25.2	
	20	35.4	30.0			15.4		25.8	35.4			18.0	32.6	26.3		
**Medications that may impact REE**																
**New or increase in dose**	8	New muscle relaxant	-	-	-	-	-	-	-	-	-	-	-	↑muscle relaxant	-	-
	12	↑ muscle relaxant	-		-	-	-	↑muscle relaxant	-		New muscle relaxant, ↑ opioids	-	-	↑muscle relaxant	-	-
	16		-				-	-	-		-		-	↑muscle relaxant	-	
	20	↑muscle relaxant	-			↑muscle relaxant		-	New muscle relaxant			↑muscle relaxants	-	↑ muscle relaxant		
**Ceased or decrease in dose**	8	↓ opioid	-	Ceased opioids	Ceased alpha-agonist, ↓opioids	-	-	-	Ceased opioid, ↓steroids	↓opioids	-	-	Ceased opioids	-	-	-
	12	↓opioid	↓opioids		↓opioids	Ceased opioids	-	Ceased opioid	-		-	-	-	-	↓opioids	Ceased opioids
	16		-					-	-		-		-	-	Ceased opioids, steroid & sympatho-mimetic amine	
	20	↓opioids	Ceased sympatho-mimetic amine			-		-	-			↓opioids	-	-		

[1]Elevated blood pressure at week 12 for 12 hours following indirect calorimetry (not meeting criteria for autonomic dysreflexia, no medical cause found)
[2]Shin cellulitis requiring antibiotics at week 8, urinary tract infection requiring antibiotics at week 16
[3]Decrease in fat-free mass of 8.8% (6.4kg) during rehabilitation
[4]Significant spasticity requiring increasing doses and numbers of muscle relaxants at week 8, 12, 16 and 20

Table 2: Mapping of barriers and enablers to study recruitment and remedial actions attempted

	Enabler	Barrier	Portfolio of remedial actions undertaken
**Screening**	Electronic medical record: screening can occur virtually	No dedicated recruitment co-ordinator or project manager separate from clinical investigator	Nurse responsible for maintaining rehabilitation unit waitlist notified Principal Investigator of upcoming admissions to enable rapid screening
		Covid disruptions to research activity	
		Need for manual screening as no automated data mining function to identify eligible patients embedded within the electronic medical record	
**Eligible**		Delays in discharge from the rehabilitation unit resulting potential recruits waiting longer in the acute wards or at other hospitals for a rehabilitation bed	Recruitment expanded to include individuals in the acute wards who were waiting for a bed in the rehabilitation unit
		Strict time frame for inclusion (4 weeks post injury)	Ethics amendment to change eligibility from within 4 weeks of injury to within 6 weeks of injury
**Approach**	Principal Investigator embedded within clinical team	Competing studies draining staff capacity to recruit to this study	Warm hand-offs from another health professional already known to the individual, and ensuring information about the study had been provided prior to initial contact by the Principal Investigator as recommended by Watson et al 2021(DOI: https://doi.org/10.1097/brs.0000000000004032)
	Research active unit	COVID-19 related staff shortages reducing ability to approach individuals for research	
	Strong multidisciplinary team support		
**Recruited**	High consent rate once approached	Burdensome frequency of data collection	Frequency of data collection reduced from fortnightly to monthly
	Research team owns equipment and responsible for use and maintenance	Complex recruitment pathway	
		Outcome measures unable to be collected remotely	
		COVID-19 related disruptions to research activity capacity	

Figure 1: Screening and recruitment flow chart

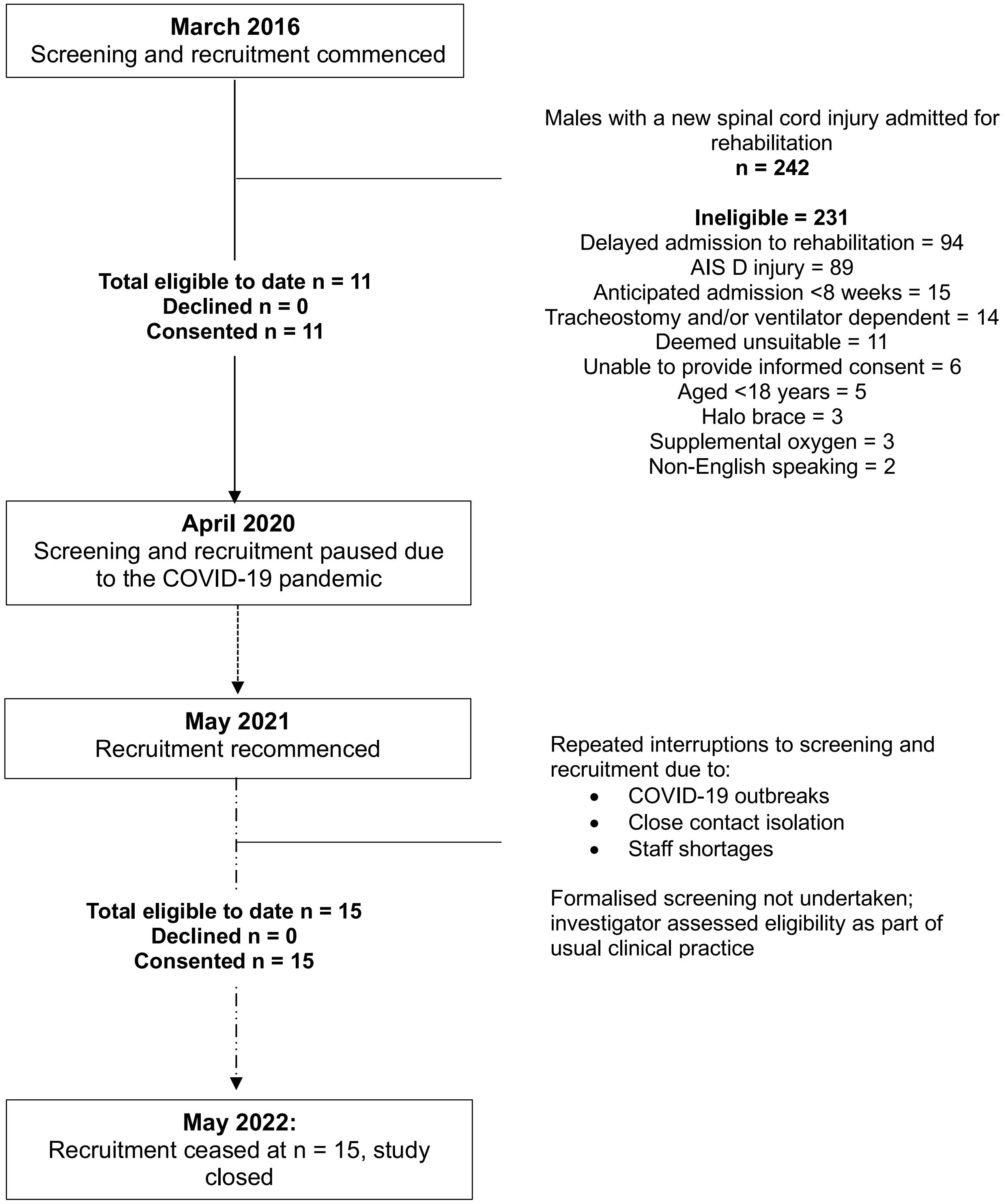

Supplement: Supplementary file 1 — Supplementary information [file 41394_2024_682_MOESM1_ESM.pdf]
